# Head and Neck Tuberculosis: A Rare Diagnosis and the Role of Surgical Biopsy and Histopathological Evaluation in Extrapulmonary Disease

**DOI:** 10.3390/pathogens14050479

**Published:** 2025-05-14

**Authors:** Carmen Aurelia Mogoantă, Andrei Osman, Alina-Maria Georgescu, Alexandra Maria Mitroi, Constantin Ioan Busuioc, Ionuţ Tănase, Ramona Cioboată, Ilona Mihaela Liliac, Ovidiu Lucian Cimpeanu, Mircea Sorin Ciolofan

**Affiliations:** 1 Department of Otorhinolaryngology, University of Medicine and Pharmacy of Craiova, 200349 Craiova, Romania; carmen.mogoanta@umfcv.ro (C.A.M.); sorin.ciolofan@umfcv.ro (M.S.C.); 2 Department of Anatomy and Embriology, University of Medicine and Pharmacy of Craiova, 200349 Craiova, Romania; 3 Doctoral School, University of Medicine and Pharmacy of Craiova, 200349 Craiova, Romania; alinamaria.georgescu@gmail.com (A.-M.G.); nutaalexandra02@gmail.com (A.M.M.); cimpeanuovidiu94@yahoo.com (O.L.C.); 4 Department of Pathology, Sfânta Maria Hospital, 011172 Bucharest, Romania; busuioc.constantin@gmail.com; 5 Department of Otorhinolaryngology, Carol Davila University of Medicine and Pharmacy, 050474 Bucharest, Romania; 6 Department of Pneumology, University of Medicine and Pharmacy, 200349 Craiova, Romania; ramona_cioboata@yahoo.com; 7 Department of Histology, University of Medicine and Pharmacy of Craiova, 200349 Craiova, Romania; ilona.liliac@umfcv.ro

**Keywords:** extrapulmonary tuberculosis, tonsillar tuberculosis, laryngeal tuberculosis, unilateral cervical lymphadenopathy, Ziehl–Neelsen staining

## Abstract

(1) Background: Extrapulmonary tuberculosis (EPTB) of the head and neck is a rare but difficult diagnosis due to mostly absent pulmonary involvement and high clinical resemblance to neoplastic or chronic inflammatory conditions. This diagnosis still poses a challenge for otorhinolaryngologists, due to non-specific symptoms and the low index of suspicion in non-endemic regions. (2) Methods: This study presents a retrospective review of nine cases of head and neck EPTB diagnosed at two regional hospitals in southern Romania. Patients presented with pharyngeal, laryngeal, or cervical lymph node involvement. All cases underwent surgical biopsies for histopathological and microbiological confirmation, followed by standard anti-tubercular therapy. (3) Results: In all nine cases, surgical biopsies were essential for the accurate diagnosis and excluded malignancy or other granulomatous diseases. Diagnostic delays were observed due to atypical clinical presentations. Integration of biopsy findings with anti-tubercular treatment resulted in favorable disease control and clinical recovery. (4) Conclusions: Head and neck EPTB requires a high index of suspicion and clinical discernment. Surgical biopsy remains a critical diagnostic tool in practice and should be considered early in the diagnostic process when encountering atypical lesions. A timely use improves diagnostic accuracy, may eliminate delays, ensures patient safety, and improves therapeutic outcomes.

## 1. Introduction

Tuberculosis (TB) continues to pose a significant global health challenge, despite continuous efforts toward its eradication [1]. According to the World Health Organization (2023), an estimated 10.6 million people developed TB globally in 2022, with 1.4 million deaths among individuals, marking a rebound in mortality after years of decline due to disruptions from the recent COVID-19 pandemic [2]. Europe, although bearing a relatively smaller burden compared to high-incidence regions like Africa or Southeast Asia, still reported approximately 230,000 TB cases in 2022 [3], with significant disparities among countries (WHO, 2023). Within the European Union (EU), the frequency of TB cases ranges significantly, from 2.6 cases per 100.000 population in Liechtenstein, to 66.2 cases per 100.000 population in Romania [Global tuberculosis report 2023. World Health Organization] [4].

Moreover, in Romania, Dolj county ranks among the top five counties with the highest incidence [5]. Romania continues to be the country with the highest TB incidence in the EU, with 50 to 60 cases per 100,000 inhabitants, nearly five times higher than the EU average. This persistent epidemiological profile underlines the relevance of TB as a national health concern in Romania, particularly in the context of diagnostic delays and underreporting in extrapulmonary disease presentations [6,7].

Pulmonary TB is by far the most common form of TB, accounting for approximately 79–85% of reported cases, while EPTB comprises 15–21%, depending on population and geographic region [8,9].

EPTB accounts for 15–20% of all TB cases in immunocompetent individuals, represents a diagnostic dilemma due to its non-specific and wide-ranging symptoms and the absence of hallmark respiratory signs [10]. Among these, head and neck TB is particularly rare, encompassing less than 1% of all TB localizations, and it is frequently misdiagnosed as malignancy or nonspecific chronic inflammation [11,12]. According to literature data, head and neck presentations of TB are infrequent, with TB cervical lymphadenopathy accounting for over 87% of cases (87–96%), laryngeal TB around 4% of cases (4.3% to 8.5%), followed by TB within the middle ear (1.4% to 3%), nasal TB (1% to 1.4%), and oral or pharyngeal TB (1.4% to 1.7%). TB may manifest in various clinical forms, mimicking neoplastic lesions or other infectious pathologies [13,14,15]. EPTB of the head and neck often presents with nonspecific and insidious symptoms, contributing to diagnostic delays. Common manifestations include persistent cervical lymphadenopathy, hoarseness, dysphagia, tonsillar hypertrophy, and slow-healing oral or pharyngeal ulcers. According to studies as those by Menon et al. [13] and Sharma & Rana [15], the average duration of symptoms prior to diagnosis can range from 4 weeks to several months, with many patients presenting late due to the absence of typical systemic symptoms like coughing or weight loss. After a proper diagnosis, symptomatic relief is generally achieved within the first 2 to 4 weeks of anti-tubercular therapy, although complete clinical resolution may take several months, particularly in cases requiring surgical intervention for abscesses or other complications.

One of the most challenging aspects of head and neck EPTB is that it may occur in the complete absence of pulmonary involvement, making the clinical diagnosis very difficult, with low suspicion towards TB-induced lesions, especially in non-endemic areas. These cases may mimic squamous cell carcinoma [16,17] or granulomatous diseases such as sarcoidosis or Wegener’s granulomatosis [18]. Therefore, histopathological examination via surgical biopsy remains a cornerstone of diagnosis, particularly when imaging and non-invasive methods yield inconclusive results.

Environmental factors, such as soil and water contamination, may play a significant role in the transmission of EPTB from animals to humans. The persistence of *Mycobacterium tuberculosis* complex bacteria in the environment, particularly in soil and water, facilitates indirect transmission routes that can affect multiple host species, including humans. Mycobacterial complexes, including *Mycobacterium bovis*, can persist in soil and water, creating reservoirs that facilitate indirect transmission. Studies have shown that these bacterial complexes can be detected in mud and water samples at wildlife aggregation points, with a significant percentage of these sites testing positive for mycobacterial presence [19]. This persistence allows for cross-species transmission, particularly in rural areas where animals congregate [19]. While environmental factors significantly contribute to the transmission of EPTB, the primary route of *M. tuberculosis* transmission remains airborne, through droplet nuclei from infectious individuals [20]. While animal-to-human transmission of TB has been acknowledged, particularly in rural areas with higher exposure to animals and livestock, the current state of research makes it difficult to discount the primary airborne route of infection as the dominant transmission pathway. Thus, although environmental reservoirs in multihost systems may contribute to TB persistence, airborne transmission of *M. tuberculosis* remains the central pathway investigated in cases of human infection [21]. This retrospective study addresses nine rare cases of EPTB in the head and neck region encountered in tertiary care centers in southern Romania. All patients were diagnosed through surgical biopsy, with no radiological or microbiological evidence of active pulmonary TB. Depending on the site, the clinical manifestation of EPTB can range from chronic behavior to acute symptoms, with rapid progression to airway obstruction. Our study highlights the clinical diversity of EPTB based on affected region, bringing awareness to the importance of an early diagnosis and treatment in order to avoid life-threatening complications.

## 2. Materials and Methods

This study is a retrospective observational case series conducted across two tertiary care centers in Romania: the Otorhinolaryngology Departments of the Emergency County Hospital of Craiova and ‘Sfânta Maria’ Clinical Hospital of Bucharest. The study period spanned ten years, from February 2015 to December 2024, and included all eligible cases of EPTB affecting the head and neck regions. The study was approved by the ethics committees of both institutions, and all procedures adhered to institutional and national guidelines on clinical research and patient confidentiality.

A total of nine patients were included in the final analysis. Inclusion criteria required a confirmed histopathological diagnosis of head and neck EPTB and absence of active pulmonary TB as demonstrated by chest radiography and microbiological testing. Patients presenting with concurrent pulmonary TB, immunodeficiency (including HIV infection), or incomplete medical records were excluded to reduce confounding and ensure diagnostic clarity. The anatomical distribution of the cases included five patients with cervical tuberculous lymphadenitis, three with laryngeal TB, and one with pharyngeal TB.

All cases underwent surgical biopsy under general anesthesia, which served as the definitive diagnostic assessment. Biopsy samples taken via aspiration by fine-needle or surgical excision were evaluated for granulomatous inflammation with or without caseating necrosis. Staining techniques like hematoxylin and eosin (HE) and Ziehl–Neelsen (ZN) were employed for histopathology research. Polymerase chain reaction (PCR) for *M. tuberculosis* was also performed when tissue volume and integrity allowed. PCR was performed using a commercially available kit targeting the IS6110 insertion sequence, commonly used for *M. tuberculosis* detection (e.g., Xpert MTB/RIF assay, Cepheid, Sunnyvale, CA, USA) because of high specificity [21].

Cases were included based on histopathological findings consistent with TB. Structured data collection was used to extract relevant variables from patient records, including age, sex, presenting symptoms, anatomical site involved, relevant imaging findings, biopsy results, therapeutic outcomes, and development of complications. Radiological evaluation through contrast-enhanced computed tomography (CT) was performed in all cases to assess lesion extent and guide surgical interventions. All patients received standard anti-tubercular therapy as per national protocols following confirmation of the diagnosis.

This study primarily utilized descriptive statistical methods to summarize the clinical characteristics and outcomes of the cohort. Clinical data were retrospectively collected from patient files archived in the hospitals’ databases. Quantitative variables were organized using Microsoft Excel (Microsoft Corporation, Redmond, WA, USA). Diagnostic imaging was sourced from the hospitals’ internal imaging libraries in standard formats such as JPEG and PNG. Selected images were processed for publication using Adobe Photoshop (Adobe Inc., San Jose, CA, USA). Only minor adjustments were applied to improve visual clarity, including standardized contrast adjustment and neutralization of skin tone variations, without altering diagnostic content. Due to the small sample size and non-comparative design, no inferential statistics were applied. No patient-identifiable data were collected, and the study adhered to the Declaration of Helsinki and ethical research standards for observational studies.

## 3. Results

This study presents the clinical characteristics, histopathological findings, and diagnostic challenges from a series of nine patients diagnosed with rare forms of EPTB affecting the head and neck regions. Given the non-specific and often misleading presentation of EPTB, these cases highlight the critical need for biopsies and histopathological diagnosis before starting standard guideline antitubercular treatment, as well as providing patients with a close follow-up to monitor local disease recurrence. The patients exhibited a wide spectrum of symptoms, including dysphonia, odynophagia, dysphagia, and weight loss, most often mimicking malignancies. Despite the absence of active pulmonary TB in all cases, definitive diagnosis was achieved through surgical biopsy, which provided essential evidence for differentiating TB from other inflammatory and neoplastic diseases.

For each site of extrapulmonary involvement, surgical biopsy was employed as the diagnostic gold standard, especially for its ability to differentiate the local masses from developing tumors. In cases of laryngeal TB, microlaryngoscopic biopsy under general anesthesia proved to be the most reliable technique, allowing for the acquisition of adequate deep tissue samples. Similarly, in pharyngeal involvement, extended excision of the affected tonsil was essential for establishing a definitive diagnosis, particularly in our case, where fine-needle aspiration cytology (FNAC) ultimately yielded negative results. For cervical lymphadenopathy, complete excision of the involved lymph nodes was found to be the most effective approach, facilitating accurate diagnosis, minimizing the risk of local recurrence, and contributing to favorable outcomes when combined with ongoing antitubercular therapy. In these cases of cervical involvement, FNAC followed by PCR targeting the IS6110 sequence of *M. tuberculosis* yielded inconclusive results due to the high predominance of necrotic material within the aspirated sample or due to the degraded quality of the deoxyribonucleic acid in the sample.

All patient outcomes were favorable, but because of atypical presentations, the correct diagnosis was delayed in all cases, with considerable life-threatening symptoms along the way, such as acute respiratory distress. Patients were constantly monitored and clinically examined for a period of two years after completing antitubercular therapy, during which no disease recurrence was observed. One case of laryngeal TB remains under ongoing surveillance. The only instance of persistent or relapsing symptoms was recorded in the patient with pharyngeal involvement. Major patient symptoms and demographic data can be visualized in Table 1.

### 3.1. Pharyngeal TB

**Figure 1:**
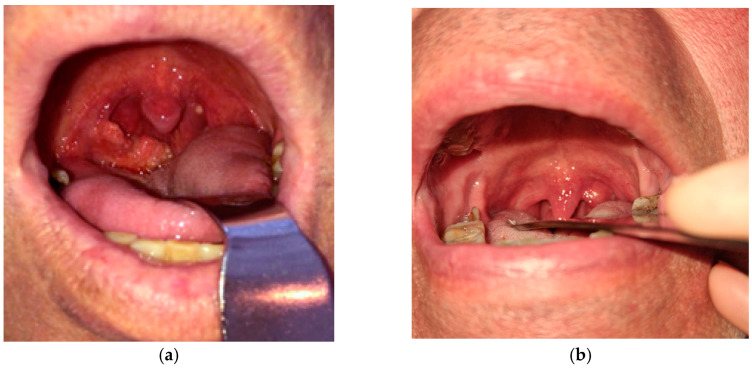
(**a**) Clinical presentation of tonsillar TB at diagnosis; (**b**) Clinical presentation of tonsillar TB after treatment.

Pharyngeal TB is an exceptionally rare [8,9] and challenging condition to diagnose, often masquerading as other infectious diseases or neoplastic processes. Given its non-specific clinical presentation, differentiating pharyngeal TB from malignancies or other chronic infections requires a high amount of suspicion, especially when the symptoms are ambiguous and the patients do not necessarily come from an endemic region. Our case of pharyngeal TB included the tonsillar region in the oropharynx (Figure 1). We report the case of a 55-year-old male patient with a history of alternating residence between urban and rural environments, potentially transitioning between areas of varying TB incidence. He was a former smoker, having stopped tobacco use 2 years prior to presentation. He had no previous history of pulmonary disease.

The patient presented with progressive dysphagia, mild to moderate odynophagia, and ipsilateral enlarged cervical lymph nodes, symptoms that can be frequently and easily mistaken for tonsillar or other pharyngeal malignancies. The patient experienced symptoms for approximately six months prior to admission, with no significant improvement despite receiving various treatments, including two courses of nonsteroidal anti-inflammatory drugs and oral amoxicillin, prescribed by the general practitioner.

Local pharyngeal presentation showed right tonsillar hypertrophy with irregular and ulcerated mucosa. Patchy yellow-whitish deposits on the tonsillar surface were likely representing caseous necrosis or local fibrinous exudate. The adjacent oropharyngeal mucosa appeared erythematous, but without gross swelling or purulence, the main lesion being mostly confined to the tonsillar area.

As flexible endoscopy did not yield any additional findings, the hypertrophic area being confined to the right tonsillar region, the main concern was differentiation between tonsillar conditions, especially ruling out a squamous cell carcinoma. In this context, imaging techniques such as ultrasonography of the neck or CT scans, though valuable, were insufficient in confirming the etiology. Neck ultrasonography revealed enlarged bilateral lymph nodes, each measuring less than 10 mm on their long axis, exhibiting features consistent with chronic reactive inflammatory changes. The chest CT scan did not reveal definitive findings suggestive of active pulmonary disease.

A significant diagnostic challenge arose when, after imaging, fine-needle aspiration cytology (FNAC) failed to provide a definitive diagnosis. In our case, the FNAC specimen was insufficient for an accurate diagnosis, as it consisted largely of acellular necrotic material. A right-sided extended tonsillectomy, including resection of the posterior tonsillar pillar due to its firm adhesion to the tonsillar tissue, was performed under general anesthesia to obtain adequate specimens for histopathological examination. The collected sample later confirmed the positive TB diagnosis.

**Figure 2:**
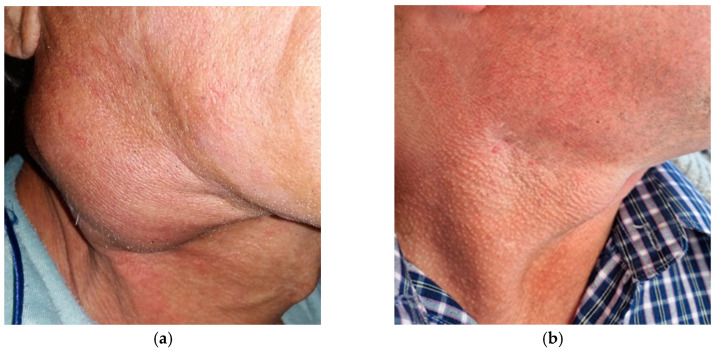
(**a**) Right cervical region during antitubercular therapy with ongoing abscess formation; (**b**) Right cervical region after surgery and antitubercular therapy.

Despite an accurate diagnosis, treatment outcomes can be complicated by related paradoxical responses, such as the development of new cervical lymphadenopathy following TB therapy initiation. In our patient, residual ipsilateral cervical lymphatic involvement and the development of a painful cervical mass needed further surgical management to excise persistent lymph nodes, as anti-tubercular treatment alone was insufficient (Figure 2). Following the surgical excision of the affected cervical lymph nodes under general anesthesia, the patient continued standard antitubercular therapy under close clinical supervision. Postoperative evolution was favorable, with gradual resolution of local inflammation and complete healing of the cervical incision site. No further signs of lymphatic reactivation or abscess recurrence were observed during follow-up.

In Romania, the treatment of non-drug-resistant TB follows the national standardized regimen, consisting of a 6-month daily therapeutic course. The initial intensive phase includes a 2-month administration of first-line antitubercular drugs: isoniazid, rifampicin, pyrazinamide, and ethambutol, followed by a 4-month continuation phase with isoniazid and rifampicin. This protocol is applied across all case categories—including new cases, relapses, and retreatments after failure—provided the isolated bacterial strain is not drug-resistant. Dosages are adjusted by weight, with a maximum daily dose of 300 mg for isoniazid, 600 mg for rifampicin, 2000 mg for pyrazinamide, and 1600 mg for ethambutol. In cases of EPTB, particularly lymphatic involvement, the continuation phase may be extended up to 12 months. Management of such cases requires interdisciplinary collaboration between pulmonologists and specialists from relevant clinical fields.

### 3.2. Laryngeal TB

Laryngeal TB is a rare but clinically significant manifestation of EPTB, easily confused with laryngeal malignancies due to its non-specific symptomatology and clinical presentation. Despite advancements in diagnostic tools, the disease remains challenging to diagnose if radiological and microbiological investigations yield inconclusive or negative results.

All three patients present in our study were middle-aged to elderly males (45, 52, and 62 years old), all smokers, who presented with symptoms typical of malignancy: dysphonia, odynophagia, dysphagia, and progressive weight loss. The symptoms were present for 6 to 9 months prior to presentation and hospital admission, with sustained hoarseness (dysphonia) and a deep, altered voice being the main complaint in all patients. All patients came from a rural background. Persistent dysphonia in smoker males will lead to a laryngeal biopsy to establish a correct diagnosis, as our current protocols indicate, even if symptoms may be consistent with the typical clinical presentation of laryngeal TB. Pulmonary imaging was unremarkable in all cases, and no history of active pulmonary involvement was recorded.

**Figure 3:**
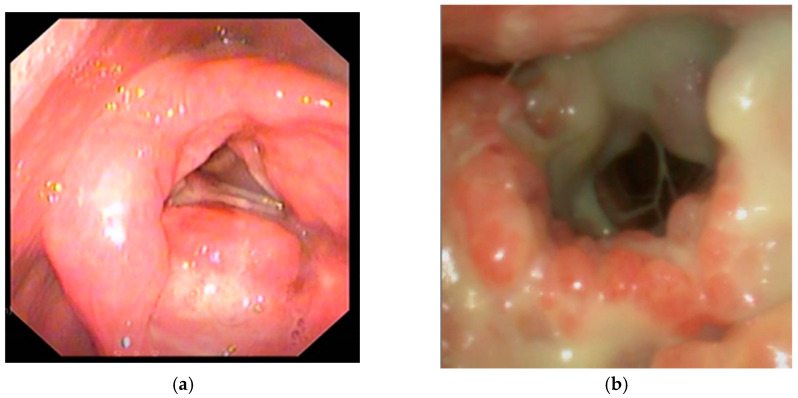
(**a**) Clinical presentation of laryngeal TB with glottic involvement; (**b**) clinical presentation of laryngeal TB with supraglottic and glottic involvement, dense exudate, and narrowed airway.

Despite local involvement, all cases demonstrated a pseudo-tumoral appearance. Endoscopic examination of the larynx revealed diffuse mucosal erythema involving the supraglottic and glottic regions, indicative of marked inflammation. The laryngeal mucosa demonstrated a nodular and irregular surface, with changes most prominent over the false vocal cords and arytenoid region, suggestive of an infiltrative disease (Figure 3). A dense purulent exudate, yellowish-white in color, was noted coating the supraglottic space and partially obscuring the glottic inlet. Multiple bulging, rounded mucosal lesions consistent with ulcerations were visualized within the supraglottic compartment of the larynx. Visualization of the true vocal cords was significantly impaired due to the presence of exudate and mucosal swelling, preventing correct assessment of vocal cord mobility. Previous biopsies were collected in two of the three patients; however, the results were inconclusive due to vastly necrotic tissue. All patients had been previously treated for chronic laryngitis for at least 6 months, and weight loss and acute respiratory distress were the main factors that determined these patients to seek medical reassessment.

In all cases, direct biopsies under general anaesthesia and microscopic guidance provided adequate tissue samples and consequently definitive histopathological evidence of TB. Two patients required the placement of a tracheostomy tube to manage acute respiratory distress prior to biopsy sampling.

Following biopsy and diagnostic confirmation, all patients received standard anti-TB therapy (isoniazid, rifampicin, pyrazinamide, and ethambutol for two months, followed by isoniazid and rifampicin for four months) per the national TB treatment guidelines. Post-treatment follow-ups revealed complete resolution of laryngeal lesions and symptoms. Patients were successfully decannulated two months after initiation of antitubercular therapy, and the tracheal stomas closed by secondary intention with good healing. The patients were also monitored for cervical masses and clinically evaluated for two years, but did not develop noticeable lesions. One patient is still under clinical surveillance.

### 3.3. TB Lymphadenitis

**Figure 4:**
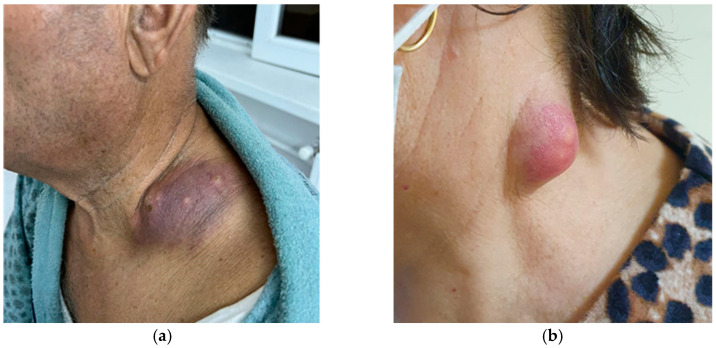
(**a**) Clinical presentation of TB lymphadenitis in a male patient—level IV cervical lymph node station; (**b**) clinical presentation of TB lymphadenitis in a female patient—level III cervical lymph node station.

We report five cases that included patients aged 3 to 76 years, with a male predominance (4:1) and a mix of rural and urban backgrounds (2:3). Patients presented with unilateral cervical masses, two of which were complicated by localized suppuration suggestive of abscess formation. Symptoms, including pain, local congestion, and progressive enlargement of the neck masses, started approximately one month prior to presentation, although one patient reported a six-month history of progressive swelling, indicating a prolonged diagnostic delay. Descriptive clinical notes in our cases pointed to local involvement of the third and fourth cervical lymph node regions with elevated, dome-shaped masses with tense overlaying skin that had a violaceous to purplish hue, suggesting underlying inflammation or ischemia. Presence of central yellow pustular points in the abscess forming cases, suggesting impending or active fistulization as per secondary infections (Figure 4).

Despite these findings, flexible endoscopy and chest CT scans were unremarkable in all cases, with no suspect pharyngeal, laryngeal, or pulmonary lesions present. Ultrasound examination revealed ill-defined, heterogeneous lymph nodes in close proximity to the sternocleidomastoid muscle, with some demonstrating features suggestive of necrosis. Considering the clinical uncertainty and overlap with malignancies or other types of infections, surgical lymph node excision was performed in all cases. Lymphadenectomy under general anesthesia was preferred over FNAC, which, in the cases with abscess formation, yielded inconclusive results due to the presence of necrotic cells.

Surgical intervention and lymph node excision and biopsy also played a therapeutic role, particularly in cases with abscess formation and necrosis extending to the skin, where drainage and complete excision prevented the further spread of infection. Postoperative wound care included daily sterile dressing, broad-spectrum antibiotics, and analgesics to ensure proper healing. After diagnosis confirmation, all patients were subsequently started on standard anti-TB therapy as per national treatment protocols. During follow-up assessments conducted while patients were undergoing antitubercular treatment, no signs of recurrent cervical lymphadenopathy or local disease progression were noted.

All patients exhibited favorable outcomes with complete resolution of symptoms. Follow-up over a period of two years confirmed the absence of local recurrence, highlighting the importance of early surgical intervention in complicated cases of TB lymphadenitis

### 3.4. Histopathological Diagnostic Findings

In our experience, FNAC did not yield adequate tissue samples to enable a successful PCR test for *M. tuberculosis*. In all presented cases, we relied on a histopathological diagnosis. The HE and ZN staining techniques were employed for the analyzed samples.

**Figure 5:**
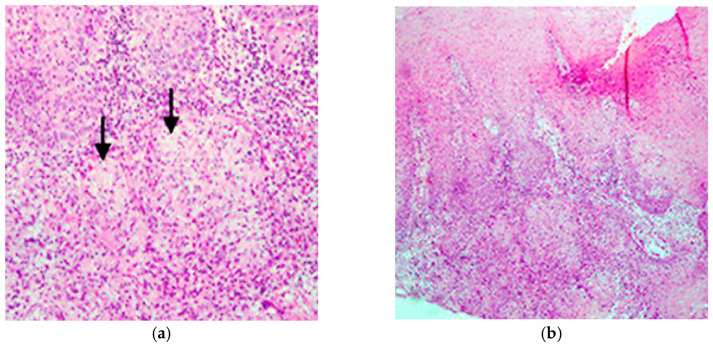
(**a**) Moderate-power HE. Granulomatous inflammation within lymphoid tissue, two centrally placed arrows highlight epithelioid granulomas, composed of elongated, pale-staining epithelioid histiocytes surrounded by a rim of mononuclear lymphocytes; (**b**) Low-power HE. Overview of the mucosal architecture and submucosal lymphoid aggregates. Extensive granulomatous inflammation, occupying the subepithelial tissue. The granulomas are less distinct at this magnification, but areas of caseating necrosis are evident in the deeper zones of the tissue.

Histopathological evaluation of pharyngeal, laryngeal, and cervical lymph node biopsies revealed characteristic features of TB infection. HE stained sections consistently demonstrated granulomatous inflammation, characterized by epithelioid cell aggregates, surrounded by peripheral lymphocytic collars—a typical immune response in TB. Notably, several biopsy specimens exhibited central areas of caseous necrosis, appearing as amorphous, acellular zones with pale staining, further supporting a tuberculous etiology. Although Langhans-type multinucleated giant cells were not universally observed in all fields, their presence was confirmed in deeper tissue levels, along with scattered lymphoid aggregates and reactive hyperplasia (Figure 5).

**Figure 6:**
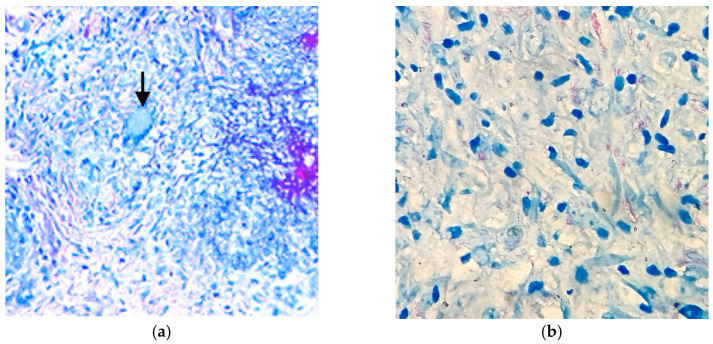
(**a**) Moderate-power ZN staining. The arrow points to a necrotizing granulomatous lesion with AFB. Rod-shaped, red-staining bacilli, consistent with *M. tuberculosis*, embedded within a background of pale blue inflammatory cells and necrotic debris. The surrounding tissue architecture shows poorly organized granulomas, with scattered epithelioid histiocytes and lymphocytes; (**b**) High-power ZN staining. Detailed view of the cellular inflammatory infiltrate and numerous AFB. Multiple curved, red bacilli are clearly visible scattered among the blue-stained inflammatory background, composed predominantly of macrophages, histiocytes, and occasional lymphocytes. Some bacilli appear to be intracellular, within macrophages—an important feature of *M. tuberculosis* pathology.

ZN staining provided microbiological confirmation by highlighting acid-fast bacilli (AFBs), appearing as red, curved rods against a blue background. In one specimen, AFBs were noted both extracellularly and intracellularly within macrophages, confirming the pathogen’s intraphagocytic localization (Figure 6). In areas with a higher bacillary load, granuloma architecture was less well-defined, suggesting either early-stage lesion development or reduced immune containment, possibly in the context of systemic immunosuppression.

## 4. Discussion

Up to date, TB remains a significant differential diagnosis to make in the field of head and neck surgery, especially in regions with high disease prevalence [22], Romania being one of these regions. Although primarily a pulmonary disease, TB can manifest in the head and neck region, often presenting a diagnostic challenge due to its varied and sometimes atypical clinical presentations.

The most common form of head and neck TB is the involvement of local lymph nodes. Patients present with enlarged cervical lymph nodes that grow slowly, seldom without any local inflammation signs. Other areas in the head and neck can also be affected, including the larynx, oropharynx, and salivary glands [23].

The incidence of head and neck TB is higher in developing countries, where TB continues to be a significant public health concern [3]. According to literature data, the age of affected patients ranges from as young as 7 months to 74 years. In comparison, our documented cases included patients between 3 and 76 years of age [24]. Patients were equally distributed among rural and urban areas, but mostly, analyzing their history, they moved between geographic areas and from low to high-density living areas.

Studies point out that enlarged cervical lymph nodes are the most common manifestation, accounting for a significant proportion of cases in various studies, ranging from 77% to 84.17% of cases [25]. The results of a meta-analysis showed that EPTB represents almost 15% of TB cases, and approximately 10–35% of cases can be found in the head and neck region, most frequently in lymph nodes (87.9%), the other most common location being the larynx [23,26,27].

In some studies, TB of the head and neck was found in patients with a history of migration to and from high TB burden areas or in those with immunosuppressive conditions [28], which is also seen in our Romanian cohort, as all our patients moved into dense urban areas from low-density rural areas 5 to 7 years previous to diagnosis [28]. For the most common site for TB in the head and neck region, the cervical lymph nodes, clinical insights often point to neck swelling, and frequently, the diagnosis can be made using fine needle aspiration and cytology exams [29,30]. FNAC and the histopathological exam are commonly used, if not gold standard, diagnostic tools [29,31]. Treatment typically involves standard antitubercular therapy, which is effective in most cases, although multidrug-resistant TB can complicate treatment [32,33,34].

Recent studies also highlight that delayed diagnosis leads to complications such as fistula formation or chronic sinus tracts, reinforcing the need for an early, aggressive diagnostic approach in cases of unexplained cervical lymphadenopathy [35].

Laryngeal TB is another notable manifestation, though less common than enlarged cervical lymph nodes. It can present with symptoms such as dysphonia and dysphagia [22,36]. TB is the main cause of granulomatous inflammatory disease of the larynx, followed by syphilis, amyloidosis, granulomatosis with polyangiitis, actinomycosis, and sarcoidosis [37,38].

Diagnosing laryngeal TB presents unique difficulty due to its rarity and the similarity in symptoms to other laryngeal conditions, particularly laryngeal carcinoma [39]. This condition is a form of EPTB that affects less than 1% of all TB cases, often leading to misdiagnosis and delayed treatment. The clinical presentation of laryngeal TB is non-specific, often displaying the characteristics of laryngeal carcinoma due to similar symptoms and laryngoscopic findings, such as ulcerations and mass-like lesions [40]. Laryngoscopy is crucial for visualizing laryngeal lesions, and biopsies are essential for histopathological confirmation. Direct or microscopic laryngoscopy is preferred. The presence of granulomas, giant cells, and caseous necrosis in samples taken is indicative of TB [39,41].

Studies have reported that delayed or misdiagnosed cases of laryngeal TB can lead to progressive airway compromise and irreversible laryngeal fibrosis, resulting in permanent voice changes or airway obstruction [42]. This reinforces the necessity of obtaining biopsies in laryngeal lesions, even when TB is not the primary clinical suspicion.

Laryngeal TB is commonly associated with pulmonary TB, and is easily mistaken for laryngeal carcinoma, especially since common symptoms are usually reported by patients, such as hoarseness for several months, dysphagia, and weight loss [43].

Given the ease of misdiagnosing TB for carcinoma, a diagnosis of certainty will imply a biopsy of the lesion followed by a histopathological examination [12]. There have been instances where, upon examination of the larynx, polypoid-like changes were seen, as per one of our laryngeal TB cases [15,44,45,46]. Histopathological exam traditionally exhibits granulomas with a central area of caseation surrounded by chronic inflammatory infiltrates and Langhans multinucleated giant cells [47].

Clinical presentation varies from non-specific inflammatory appearance to ulcerative or exophytic lesions. Its inconsistent aspect and capability to mimic a malignant process make the diagnosis difficult and delay the start of targeted treatment [21]. Various studies found dysphonia as the most common symptom, followed by dyspnea and odynophagia [37,48]. Secondary laryngeal TB is described in up to 15–37% of TB, but primary laryngeal development is exceptionally rare, with an incidence of only 1% [49]. The main treatment is primarily medical and has excellent results, but long-term surveillance is necessary as local complications can occasionally occur at the level of the larynx. Surgical procedures are required only in those cases where the airway is compromised [50]

Moreover, existing literature suggests that laryngeal TB is increasingly being identified in immunocompetent individuals, especially post-COVID-19, contrasting with historical cases where it was predominantly seen in immunocompromised populations. This further supports the need for routine histopathological exams of suspicious laryngeal lesions, regardless of patient immune status or pulmonary TB history [51].

TB can also affect the oropharynx and oral cavity, though these presentations are rare and often misdiagnosed as other potential malignancies due to its hypertrophic presentation [11]. The involvement of the salivary glands and deep neck spaces is less common but documented in some cases, as literature points out, thus, it should not be completely ruled out in chronic, poorly documented cases of salivary gland pathology [52,53].

Tonsillar TB, one of the rarer forms of extrapulmonary TB, was confirmed only through excisional biopsy and histopathology exam. Differential diagnosis led us to a large array of pathologies, such as tonsillar tumours, granulomatous lesions (sarcoidosis, actinomycosis), ulceronecrotic tonsillitis, and different preneoplastic lesions. Typical lesions of oral TB are usually irregular, accompanied by painful ulcers that steadily increase in size. Commonly found in the oral region, easily traumatised surfaces may simply be mistaken for traumatic ulcers or carcinomas, as per our case [15,54].

Differentiating between TB and sarcoidosis in sampled biopsies is a significant challenge due to their overlapping characteristics. Both diseases may resemble each other from a clinical, radiological, and even histopathological point of view. Both conditions are granulomatous diseases, which complicates the histopathological differential diagnosis. Both TB and sarcoidosis present with granulomatous inflammation. TB typically shows caseating granulomas, while sarcoidosis is characterized by non-caseating granulomas. However, necrotizing sarcoidosis can mimic TB, making histopathological differentiation more difficult without additional clinical context or further testing [55,56]. Morphometric analysis may also assist with differential diagnosis. A study highlighted the use of morphometric analysis to differentiate granulomas, noting that sarcoidosis granulomas have increased lymphocytes and fibroblasts, while TB granulomas have more granulocytes and epithelioid cells [55].

Molecular and culture tests have been proven useful. The Xpert MTB/RIF assay and QuantiFERON-TB Gold tests have shown high specificity and sensitivity in distinguishing TB from sarcoidosis. These tests can detect mycobacterial DNA or immune response to TB antigens, which are absent in sarcoidosis [57]. The disadvantages of PCR testing are that an adequate FNAC sample or tissue biopsy is always required, and QuantiFERON-TB Gold tests do not distinguish active from latent TB.

The differential diagnosis debate has efficiently transitioned into serum biomarkers. The neutrophil to lymphocyte ratio and platelet to lymphocyte ratio have been investigated as potential biomarkers. These markers are significantly higher in TB compared to sarcoidosis, providing a non-invasive diagnostic aid [58].

In patients where non-invasive methods are inconclusive, surgical biopsies remain a definitive and most valuable diagnostic tool. Histological examination of completely excised lymph nodes or other pharyngeal or laryngeal lesions can confirm TB through the presence of mycobacteria or caseating granulomas, thus, the careful surgical gesture that leads to a correct diagnosis remains a near-gold standard for confusing cases [59].

The AFB in macrophages can be a useful indicator of TB, but its reliability varies depending on the context and method of detection. AFBs are a hallmark of *M. tuberculosis* infection, and their detection in macrophages is a critical component of TB diagnosis. However, the sensitivity and specificity of AFB detection can be influenced by the site of infection, the method of sample collection, and the staining techniques used. *M. tuberculosis* is an intracellular pathogen that primarily infects macrophages, leading to the formation of granulomas in the lungs. AFBs can be visualized within these macrophages, making their presence a potential indicator of TB infection [60]. The relationship between macrophages and *M. tuberculosis* is complex, with macrophages playing a central role in the host’s immune response to TB. This relationship underscores the importance of detecting AFBs within macrophages as part of the diagnostic process [61,62]. The ZN staining method is commonly used to detect AFBs in various samples, including buffy coat and bone marrow smears. However, the sensitivity of AFB detection using this method can be limited, with detection rates varying significantly across different studies and sample types [63]. In cases of extrapulmonary TB, such as lymphadenitis, the detection of AFBs can be challenging. Studies have shown that the sensitivity of conventional methods may not exceed 40% in these cases, highlighting the need for alternative diagnostic approaches [64].

In confusing cases, uncertainty lingers even after the microscopic evaluation of biopsies, particularly when the biopsy samples are taken only from the superficial mucosal layers. In order to avoid this pitfall, multiple and deeper biopsies must be taken from the site of interest. Another aspect that clinicians must not disregard is the coexistence of TB and carcinoma in the same patient, several cases being reported in the literature [20,33] regarding this particularly rare situation.

Despite the clinical importance of the presented cases, which address an uncommon disease, EPTB, several limitations should be taken into account. The small number of cases (n = 9), though reflective of the rarity of head and neck TB, restricts the extent to which our findings can be applicable to all populations. While the data were collected from two major hospitals in southern Romania, the absence of a population-level control group restricts our ability to draw definitive conclusions regarding potential transmission dynamics, including interhuman spread, zoonotic exposure, poor hygiene, or latent TB reactivation in the aftermath of other pandemics. This limitation is especially pertinent given that many patients in our study reported alternating residence between rural and urban environments, complicating epidemiological attribution.

Another significant limitation is the restricted use of molecular diagnostic tools. While PCR remains an essential tool in recent protocols for diagnosing TB [65], its application was hindered in all cases by the inadequate samples obtained via FNAC. This likely reflects the extended delays in patient presentation and biopsy collection [66], as symptoms were typically nonspecific and progressed for months prior to a correct diagnosis.

## 5. Conclusions

EPTB of the head and neck remains a rare but diagnostically challenging entity, often resembling malignancies or chronic inflammatory conditions. In this retrospective analysis of nine cases from high TB-incidence regions in Romania, we demonstrated that surgical biopsies are still an essential tool for achieving diagnostic accuracy, particularly in cases where imaging, FNAC, and other non-invasive tests yield inconclusive results. The most important feature of our study is that all investigated patients lacked active pulmonary TB involvement, highlighting situations where the need for elevated clinical vigilance is recommended even in the absence of respiratory symptoms.

The necessity for a multidisciplinary approach involving otorhinolaryngologists, infectious disease specialists, and pathologists cannot be overstated. Early surgical biopsy of suspect lesions plays a crucial role in guiding timely antitubercular therapy, ultimately improving patient outcomes and eliminating possible life-threatening complications.

## Figures and Tables

**Table 1:** Patient demographics and clinical symptoms.

Patient	Age	Gender	Residence	Clinical Symptoms	Symptom Duration	Site
1	55	M	Urban/Rural	Dysphagia, odynophagia, cervical lymphadenopathy	6 months	Pharyngeal (tonsil)
2	45	M	Rural	Dysphonia, odynophagia, weight loss	6–9 months	Laryngeal
3	52	M	Rural	Dysphonia, odynophagia, weight loss	6–9 months	Laryngeal
4	62	M	Rural	Dysphonia, odynophagia, weight loss	6–9 months	Laryngeal
5	3	M	Urban	Cervical mass, pain, local inflammation	1 month	Cervical lymph nodes
6	55	F	Urban	Cervical mass, pain, local inflammation	1 month	Cervical lymph nodes
7	30	M	Rural	Cervical mass, pain, local inflammation	1 month	Cervical lymph nodes
8	59	F	Urban/Rural	Cervical mass, abscess formation, discoloration, pain	1 month	Cervical lymph nodes
9	76	M	Rural	Cervical mass, abscess formation, discoloration, pain	6 months	Cervical lymph nodes

## Data Availability

The data presented in this study are available on request from the corresponding authors.

## Abbreviations

The following abbreviations are used in this manuscript:

EPTB	Extrapulmonary Tuberculosis
TB	Tuberculosis
EU	European Union
ZN	Ziehl–Neelsen
FNAC	Fine Needle Aspiration Citology
CT	Computed Tomography
HE	Hematoxylin and Eosin
AFBs	Acid-fast Bacilli
